# Computed tomography‐determined skeletal muscle density predicts 3‐year mortality in initial‐dialysis patients in China

**DOI:** 10.1002/jcsm.13331

**Published:** 2023-09-18

**Authors:** Ming‐jie Sheng, Jing‐yuan Cao, Shi‐mei Hou, Min Li, Yao Wang, Qiang Fang, A‐feng Miao, Min Yang, Shu‐su Liu, Chun‐hong Hu, Cui‐lan Liu, Shi‐yuan Wang, Jing Zheng, Jing‐jie Xiao, Xiao‐liang Zhang, Hong Liu, Bi‐cheng Liu, Bin Wang

**Affiliations:** ^1^ Department of Nephrology, Zhong Da Hospital Southeast University School of Medicine Nanjing China; ^2^ Department of Nephrology The Affiliated Kunshan Hospital of Jiangsu University Kunshan China; ^3^ Department of Nephrology The Affiliated Taizhou People's Hospital of Nanjing Medical University, Taizhou School of Clinical Medicine, Nanjing Medical University Taizhou China; ^4^ Department of Nephrology The First People's Hospital of Changzhou Changzhou China; ^5^ Department of Nephrology The Affiliated Hospital of Yangzhou University, Yangzhou University Yangzhou China; ^6^ Department of Epidemiology and Health Statistics Southeast University School of Public Health Nanjing China; ^7^ Department of Geriatrics, Zhong Da Hospital Southeast University School of Medicine Nanjing China; ^8^ Covenant Health Palliative Institute Edmonton Canada

**Keywords:** Computed tomography, Dialysis, First lumbar vertebra level, Prognosis, Skeletal muscle quality

## Abstract

**Background:**

Skeletal muscle mass and quality assessed by computed tomography (CT) images of the third lumbar vertebra (L3) level have been established as risk factors for poor clinical outcomes in several illnesses, but the relevance for dialysis patients is unclear. A few studies have suggested a correlation between CT‐determined skeletal muscle mass and quality at the first lumbar vertebra (L1) level and adverse outcomes. Generally, chest CT does not reach beyond L1. We aimed to determine whether opportunistic CT scan (chest CT)‐determined skeletal muscle mass and quality at L1 are associated with mortality in initial‐dialysis patients.

**Methods:**

This 3‐year multicentric retrospective study included initial‐dialysis patients from four centres between 2014 and 2017 in China. Unenhanced CT images of the L1 and L3 levels were obtained to assess skeletal muscle mass [by skeletal muscle index, (SMI), cm^2^/m^2^] and quality [by skeletal muscle density (SMD), HU]. Skeletal muscle measures at L1 were compared with those at L3. The sex‐specific optimal cutoff values of L1 SMI and L1 SMD were determined in relation to all‐cause mortality. The outcomes were all‐cause death and cardiac death. Cox regression models were applied to investigate the risk factors for death.

**Results:**

A total of 485 patients were enrolled, of whom 257 had both L1 and L3 images. Pearson's correlation coefficient between L1 and L3 SMI was 0.84 (*P* < 0.001), and that between L1 and L3 SMD was 0.90 (*P* < 0.001). No significant association between L1 SMI and mortality was observed (*P* > 0.05). Low L1 SMD (*n* = 280, 57.73%) was diagnosed based on the optimal cutoff value (<39.56 HU for males and <33.06 HU for females). Multivariate regression analysis revealed that the low L1 SMD group had higher risks of all‐cause death (hazard ratio 1.80; 95% confidence interval 1.05–3.11, *P* = 0.034) and cardiac death (hazard ratio 3.74; 95% confidence interval 1.43–9.79, *P* = 0.007).

**Conclusions:**

In initial‐dialysis patients, there is high agreement between the L1 and L3 measures for SMI and SMD. Low SMD measured at L1, but not low SMI, is an independent predictor of both all‐cause death and cardiac death.

## Introduction

Sarcopenia is defined as a generalized, progressive and age‐related skeletal muscle disorder characterized by the loss of skeletal muscle mass, quality and function.^1^ It is associated with adverse outcomes such as debility, increased health care costs and increased mortality.^1^, ^2^ It is suggested as a possible prognostic factor in various types of cancer^3^, ^4^, ^5^, ^6^ and other non‐cancer diseases, such as heart failure^7^ and chronic obstructive pulmonary disease.^8^

Patients with chronic kidney disease, especially those on dialysis, are more prone to sarcopenia than healthy people of the same age due to factors such as decreased nutrient intake, protein wasting and metabolic derangement.^1^, ^2^, ^9^ Studies have shown that dialysis patients with sarcopenia have poorer outcomes than those without sarcopenia.^9^ However, as such studies are influenced by factors such as different races, techniques and co‐morbidities,^1^ a consensus on the diagnosis of sarcopenia and its impact on prognosis in dialysis patients has not been reached to date.^10^ Therefore, a stable and convenient method for assessing skeletal muscle mass and quality is needed for early identification of sarcopenia in dialysis patients.

There is increasing interest in using opportunistic CT to evaluate sarcopenia. CT‐based assessment makes it possible to quantitate skeletal muscle mass [by skeletal muscle index (SMI), cm^2^/m^2^] and quality [by skeletal muscle density (SMD) HU] objectively.^11^ SMI represents the height‐corrected skeletal muscle area. SMD represents the mean radiodensity of the measured cross‐sectional skeletal muscle area. Many reports on cancer have shown that low SMI (reflecting low amounts of lean mass) and/or low SMD (reflecting higher amounts of fat infiltration into muscle) of the third lumbar vertebra (L3) are associated with adverse outcomes such as higher risks of death, postsurgical complications and a longer length of hospital stay.^4^, ^5^, ^6^ Because L3 (obtained from abdominal CT) is not always included in opportunistic CT scans (chest CT), CT of the first lumbar vertebra (L1) might be a good substitute for muscle assessment.^12^, ^13^ Some studies have indicated that low L1 SMI and/or SMD are associated with adverse outcomes in diseases such as non‐small cell lung cancer^14^ and acute myeloid leukaemia.^15^

Recently, Wu et al. found that CT‐assessed sarcopenia obtained using the psoas muscle index was associated with poor peritoneal dialysis (PD) survival and overall survival in PD patients.^16^ Sabatino et al. found that CT‐assessed low skeletal muscle area is associated with all‐cause mortality in haemodialysis (HD) patients.^17^ The present studies on the utility of CT for assessing sarcopenia have focused on L3 and skeletal muscle mass assessment,^16^, ^17^ and whether CT‐assessed skeletal muscle mass and quality at the L1 level are correlated with the mortality of dialysis patients remains unclear. Therefore, we performed a multicentre retrospective study to determine CT‐assessed skeletal muscle mass and quality at L1 and their associations with all‐cause mortality and cardiac mortality in initial‐dialysis patients.

## Materials and methods

### Study design and participants

We conducted a multicentre, retrospective study of patients on dialysis in the department of nephrology and blood purification centres from January 2014 to December 2017 at four centres, namely, Zhong Da Hospital, the First People's Hospital of Changzhou, the First People's Hospital of Yangzhou and the Affiliated Taizhou People's Hospital of Nanjing Medical University in China. To be considered for inclusion, patients with end‐stage renal diseases had to range in age from 18 to 75 years and must have started to undergo HD or PD. Unenhanced CT scans of the chest and/or abdomen usually performed as a routine preoperative examination hospitalized, including L1 and/or L3, were taken within 1 month before or after the commencement of routine dialysis. Patients who had malignancy, liver failure, respiratory failure, inflammatory bowel disease, Alzheimer's disease, Guillain–Barre syndrome, transplanted kidneys and amputated limbs were excluded. This study was approved by the medical research ethics board of the Zhong Da Hospital (2022ZDSYLL003‐P01) and was registered in the Chinese Clinical Trial Registry (ChiCTR2300068453).

### Measurements of skeletal muscle mass and quality indicators

Single‐slice transverse CT images at L1 and L3 were determined to quantify skeletal muscle mass and quality.^18^ CT scanners from General Electric (GE) (128‐slice spiral CT scanners from GE discovery CT, 128‐slice spiral CT scanners from GE optima CT660, and 256‐slice spiral CT scanners from GE revolution CT) and Siemens (64‐slice spiral CT scanners from SOMATOM sensation CT, SOMATOM definition flash CT, and SOMATOM definition AS CT, and 96‐slice spiral CT scanners from SOMATOM force CT) were used. Image analysis was conducted with ImageJ (version 1.46).^19^

The value of HU represents the radiodensity. Tissues were measured by using a threshold for HU of −29 to +150 for skeletal muscle^20^ (*Figure* S1). The mean radiodensity of the whole muscle area at the L1 and L3 cross‐sectional areas was selected for analysis of SMD (indicator used to quantify skeletal muscle quality, HU). SMI (cm^2^/m^2^) was represented by the skeletal muscle area corrected by body height squared.^21^

### Measurements of covariates

Baseline characteristics, including age, sex, height, weight, dialysis methods (HD or PD), history of smoking, clinical laboratory results [haemoglobin (Hb), platelet (PLT), albumin (ALB), serum creatinine (SCr) and cystatin C (CysC)], co‐morbidities (diabetes, hypertension and coronary heart disease) and medication history (iron agent, erythropoietin and compound ɑ‐keto acid tablets), were retrieved from the electronic medical record (EMR). Clinical laboratory results closest to and prior to the date of the CT scans and routine dialysis were collected.

### End points

All patients were followed up from the date of the CT scan until death or the end of the three‐year follow‐up. Ultimately, the database of the endpoints was established by EMR and telephone consultation. In this study, the endpoints were all‐cause death and cardiac death.

### Statistical analysis

Analyses were performed using SPSS version 26.0 (IBM Corp.). A two‐sided test *P* < 0.05 was performed to define statistical significance. In the analyses, normally distributed continuous variables are presented as the mean and standard deviation (SD), non‐normally distributed continuous variables are presented as the median and interquartile range (IQR), and categorical data are presented as absolute numbers and percentages (%). When appropriate, the chi‐squared test, *t* test, or Wilcoxon *Z* test was applied to compare clinical variables.

The correlations of SMI and SMD between L1 and L3 were investigated by Pearson correlation analysis. The time‐dependent receiver operating characteristic (ROC) curve was used to determine the sex‐specific optimal cutoff values of SMI and SMD in relation to all‐cause mortality.^10^ Cox regression analysis was used to determine the associations of L1 SMD/SMI (low and normal), sex, age, BMI, smoking history (yes and no), past history (diabetes, hypertension and coronary heart disease), medication history (iron agent, erythropoietin and compound α‐keto acid tablets), and laboratory results (Hb, PLT, ALB and SCr/CysC) with all‐cause death and cardiac death separately. Hazard ratios (HRs) and corresponding 95% confidence intervals (CIs) were calculated.

Kaplan–Meier survival analysis was performed to assess the contribution of L1 SMD to all‐cause mortality. Overall survival was defined as the interval from the date of the CT scan to all‐cause death. To predict the occurrence risk of all‐cause death, factors that were significant in multivariate analysis (*P* < 0.05) were selected to establish the nomograms to explore the 1‐, 2‐, and 3‐year survival rates.

## Results

### Baseline characteristics

**Figure 1:**
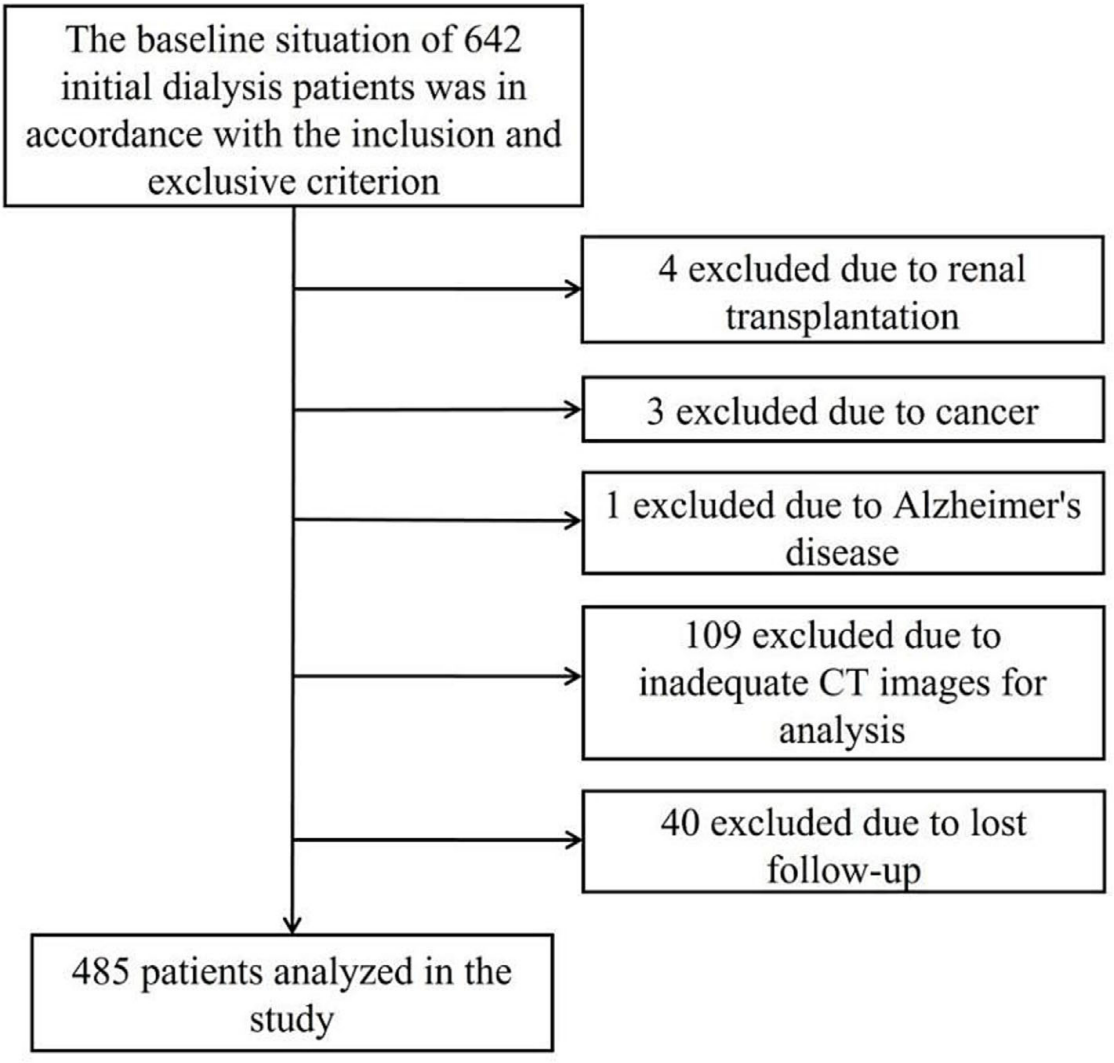
Flowchart of patient selection. From 642 initial dialysis patients, 485 were eligible for inclusion. According to the 3‐year follow‐up, patients were excluded due to renal transplantation (*n* = 4), cancer (*n* = 3), Alzheimer's disease (*n* = 1), inadequate CT images for analysis (*n* = 109) and lost follow‐up (*n* = 40).

A total of 485 patients with L1 level images were eligible for the study (*Figure* 1). The median (IQR) age involved was 57 (46, 65) years, 303 (62.47%) patients were male and the median (IQR) BMI was 23.2 (21.1, 25.8) kg/m^2^. During the 3‐year follow‐up period, 89 (18.35%) patients died, and 46 (9.48%) of whom died of cardiac reasons (*Table* 1).

**Table 1:** Baseline characteristics of the study population

Characteristic		SMD^a^		
	Low SMD^b^ (*n* = 280)	Normal SMD^c^ (*n* = 205)	Overall (*n* = 485)	*P‐*value
Age, year, median (IQR^d^)	62 (51, 68)	49 (39, 60)	57 (46, 65)	<0.001
Sex, *n* (%)				0.84
Male	176 (62.86%)	127 (61.95%)	303 (62.47%)	
Female	104 (37.14%)	78 (38.05%)	182 (37.53%)	
Height, m, median (IQR)	1.67 (1.60, 1.72)	1.67 (1.60, 1.72)	1.67 (1.60, 1.72)	0.91
Weight, kg, median (IQR)	65.8 (59.6, 74.0)	61.0 (54.3, 70.0)	65.0 (57.0, 72.0)	<0.001
BMI^e^, kg/m^2^, median (IQR)	24.2 (21.5, 26.7)	22.3 (20.3, 24.1)	23.2 (21.1, 25.8)	<0.001
Smoking history, *n* (%)	81 (29%)	53 (26%)	134 (28%)	0.45
Dialysis methods, *n* (%)				<0.001
Haemodialysis	250 (89%)	148 (72%)	398 (82%)	
Peritoneal dialysis	30 (11%)	57 (28%)	87 (18%)	
Past history, *n* (%)				
Type 2 diabetes	133 (48%)	57 (28%)	190 (39%)	< 0.001
Hypertension	254 (91%)	180 (88%)	434 (89%)	0.30
Coronary heart disease	54 (19%)	20 (10%)	74 (15%)	0.004
Medication history, *n* (%)				
Iron agent	146 (52%)	85 (41%)	231 (48%)	0.02
EPO^f^	255 (91%)	178 (87%)	433 (89%)	0.14
Compound α‐keto acid tablets	134 (48%)	106 (52%)	240 (49%)	0.42
Laboratory results				
Hb^g^, g/L, median (IQR)	82 (73, 94)	84 (74, 95)	83 (73, 95)	0.21
PLT^h^, ×10^9^/L, median (IQR)	170 (129, 231)	167 (121, 219)	168 (123, 222)	0.34
ALB^i^, g/L, mean ± SD^j^	31.7 ± 6.1	33.0 ± 5.8	32.3 ± 6.0	0.01
SCr/CysC^k^, median (IQR)	21.7 (13.3, 30.1)	20.7 (14.3, 30.1)	21.2 (13.6, 30.1)	0.47
End points, *n* (%)				
All‐cause death	70 (25.00%)	19 (9.27%)	89 (18.35%)	<0.001
Cardiac death	41 (14.64%)	5 (2.44%)	46 (9.48%)	<0.001

^a^ SMD, skeletal muscle density.

^b^ Low SMD: SMD < 39.56 Hounsfield units (HU) for males and <33.06 HU for females.

^c^ Normal SMD: SMD ≥ 39.56 HU for males and ≥33.06 HU for females.

^d^ IQR, interquartile range.

^e^ BMI, body mass index (calculated as weight in kilograms divided by height in meters squared).

^f^ EPO, erythropoietin.

^g^ Hb, haemoglobin.

^h^ PLT, platelet.

^i^ ALB, albumin.

^j^ SD, standard deviation.

^k^ SCr/CysC, serum creatinine divided by cystatin C.

The time‐dependent ROC curve based on all‐cause death was used to determine the sex‐specific optimal cutoff values. As shown in *Figure* *S2*, the optimal diagnostic cutoff values of SMD for males and females were 39.56 HU (sensitivity 76.8%; specificity 46.2%; AUC 0.621, *P* < 0.001), and 33.06 HU (sensitivity 84.8%; specificity 48.3%; AUC 0.679, *P* < 0.001), respectively. For males, there was no significant association between SMI and all‐cause death (*P* > 0.05). The low SMD group included 280 patients (57.73%), and 176 patients were male (62.86%). Patients who were older, heavier, had higher BMI, were administered HD, had a history of type 2 diabetes or coronary heart disease, had a medication history of the iron agent, and had lower ALB (*P* < 0.05) were more likely to have low SMD (*Table* 1).

### Correlations between L1 and L3 levels

**Figure 2:**
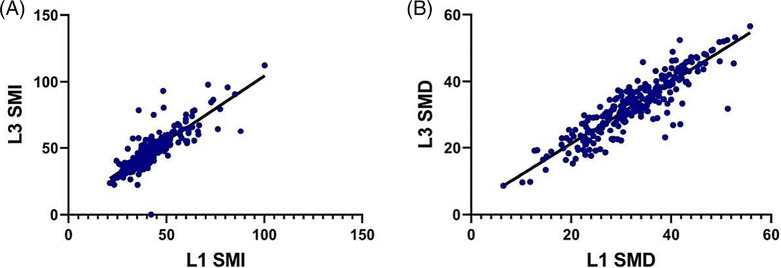
Correlations between measures at the L1 and L3 for SMI, and SMD, separately. (A) Pearson's correlation coefficient between L1 SMI and L3 SMI was 0.84 (*P* < 0.001). (B) Pearson's correlation coefficient between L1 SMD and L3 SMD was 0.90 (*P* < 0.001).

A total of 257 patients who had both L1 and L3 images were included in the Pearson correlation analysis. Pearson's correlation coefficient of SMI between L1 and L3 was 0.84 (*P* < 0.001) (*Figure* 2A). The correlation coefficient of SMD between the two CT slices was 0.90 (*P* < 0.001) (*Figure* 2B). Scatter plots of the L1 and L3 SMI and L1 and L3 SMD showed a slight spread around the lines of complete agreement, indicating high agreement.

### Risk factors for all‐cause death and cardiac death

The low SMD group had significantly higher all‐cause mortality rates (25.00%) and cardiac mortality rates (14.64%) (*P* < 0.001) than the normal SMD group (all‐cause mortality rates: 9.27%, cardiac mortality rates: 2.44%) (*Table* 1).

As shown in *Table* 2, in the univariate Cox regression analysis, low SMD, older age, HD, smoking history, history of type 2 diabetes, history of coronary heart disease and higher PLT were risk factors (*P* < 0.05). In the multivariate Cox regression analysis, low SMD (HR 1.80; 95% CI 1.05–3.11, *P* = 0.034), older age (HR 1.04; 95% CI 1.02–1.06, *P* = 0.001), smoking history (HR 1.59; 95% CI 1.03–2.44, *P* = 0.035) and history of type 2 diabetes (HR 1.64; 95% CI 1.05–2.57, *P* = 0.029) remained independent risk factors for all‐cause death. There was no significant association between SMI and all‐cause death (*P* > 0.05).

**Table 2:** Univariate and multivariate Cox regression analyses for all‐cause death

Covariables	L1 level			
	Univariate Cox regression analyses (*n* = 485)		Multivariate Cox regression analyses (*n* = 485)	
	HR (95% CI)	*P*‐value	HR (95% CI)	*P*‐value
Low SMD^a^, HU	2.94 (1.77–4.89)	<0.001	1.80 (1.05–3.11)	0.034
SMI^b^,cm^2^/m^2^	1.01 (0.99–1.03)	0.229	1.003 (0.984–1.023)	0.758
Sex (male)	1.00 (0.65–1.54)	0.994		
Age (66–75 years)	1.05 (1.03–1.08)	<0.001	1.04 (1.02–1.06)	0.001
BMI^c^, kg/m^2^	0.97 (0.91–1.03)	0.255		
Dialysis methods (haemodialysis)	2.11 (1.06–4.20)	0.034	1.34 (0.66–2.72)	0.425
Smoking history	1.87 (1.23–2.86)	0.004	1.59 (1.03–2.44)	0.035
Type 2 diabetes	2.49 (1.63–3.80)	<0.001	1.64 (1.05–2.57)	0.029
Hypertension	1.14 (0.55–2.36)	0.721		
Coronary heart disease	2.23 (1.40–3.56)	0.001	1.15 (0.70–1.91)	0.578
Iron agent	1.13 (0.75–1.71)	0.570		
EPO^d^	1.05 (0.53–2.10)	0.882		
Compound α‐keto acid tablets	0.84 (0.56–1.28)	0.426		
Hb^e^, g/L	0.99 (0.98–1.01)	0.324		
PLT^f^, ×10^9^/L	1.002 (1.000–1.004)	0.036	1.002 (1.000–1.004)	0.099
ALB^g^, g/L	0.98 (0.95–1.01)	0.254		
SCr/CysC^h^	0.995 (0.987–1.003)	0.190		

^a^ Low SMD, low skeletal muscle density: SMD <39.56 Hounsfield Units (HU) for males and <33.06 HU for females.

^b^ SMI, skeletal muscle index.

^c^ BMI, body mass index.

^d^ EPO, erythropoietin.

^e^ Hb, haemoglobin.

^f^ PLT, platelet.

^g^ ALB, albumin.

^h^ SCr/CysC, serum creatinine divided by cystatin C.

As presented in *Table* 3, low SMD, older age, smoking history, history of type 2 diabetes, history of coronary heart disease and higher PLT were found to be risk factors for cardiac death in the univariate Cox regression analysis (*P* < 0.05). In the multivariate Cox regression analysis, low SMD (HR 3.74; 95% CI 1.43–9.79, *P* = 0.007), older age (HR 1.04; 95% CI 1.01–1.08, *P* = 0.011), smoking history (HR 1.84; 95% CI 1.02–3.33, *P* = 0.043) and history of type 2 diabetes (HR 1.91; 95% CI 1.00–3.65, *P* = 0.049) were risk factors for cardiac death. There was no significant association between SMI and cardiac death (*P* > 0.05).

**Table 3:** Univariate and multivariate Cox regression analyses for cardiac death

Covariables	L1 level			
	Univariate Cox regression analyses (*n* = 485)		Multivariate Cox regression analyses (*n* = 485)	
	HR (95% CI)	*P*‐value	HR (95% CI)	*P*‐value
Low SMD^a^, HU	6.57 (2.60–16.63)	<0.001	3.74 (1.43–9.79)	0.007
SMI^b^, cm^2^/m^2^	1.01 (0.99–1.04)	0.226	1.01 (0.99–1.03)	0.711
Sex (Male)	1.50 (0.79–2.85)	0.215		
Age (66–75 years)	1.07 (1.04–1.11)	< 0.001	1.04 (1.01–1.08)	0.011
BMI^c^, kg/m^2^	1.01 (0.94–1.09)	0.745		
Dialysis methods (haemodialysis)	2.49 (0.89–6.95)	0.081		
Smoking history	2.32 (1.30–4.14)	0.005	1.84 (1.02–3.33)	0.043
Type 2 diabetes	3.52 (1.90–6.51)	<0.001	1.91 (1.00–3.65)	0.049
Hypertension	5.08 (0.70–36.83)	0.108		
Coronary heart disease	3.88 (2.15–7.02)	<0.001	1.75 (0.92–3.34)	0.087
Iron agent	1.71 (0.95–3.09)	0.076		
EPO^d^	1.24 (0.45–3.46)	0.681		
Compound α‐keto acid tablets	0.99 (0.56–1.77)	0.976		
Hb^e^, g/L	1.00 (0.98–1.01)	0.503		
PLT^f^, ×10^9^/L	1.003 (1.000–1.006)	0.031	1.003 (1.000–1.006)	0.088
ALB^g^, g/L	1.00 (0.95–1.04)	0.844		
SCr/CysC^h^	1.00 (0.98–1.01)	0.259		

^a^ Low SMD, low skeletal muscle density: SMD < 39.56 Hounsfield Units (HU) for males and < 33.06 HU for females.

^b^ SMI: skeletal muscle index.

^c^ BMI, body mass index.

^d^ EPO, erythropoietin.

^e^ Hb, haemoglobin.

^f^ PLT, platelet.

^g^ ALB, albumin.

^h^ SCr/CysC, serum creatinine divided by cystatin C.

### Association of skeletal muscle density with overall survival

**Figure 3:**
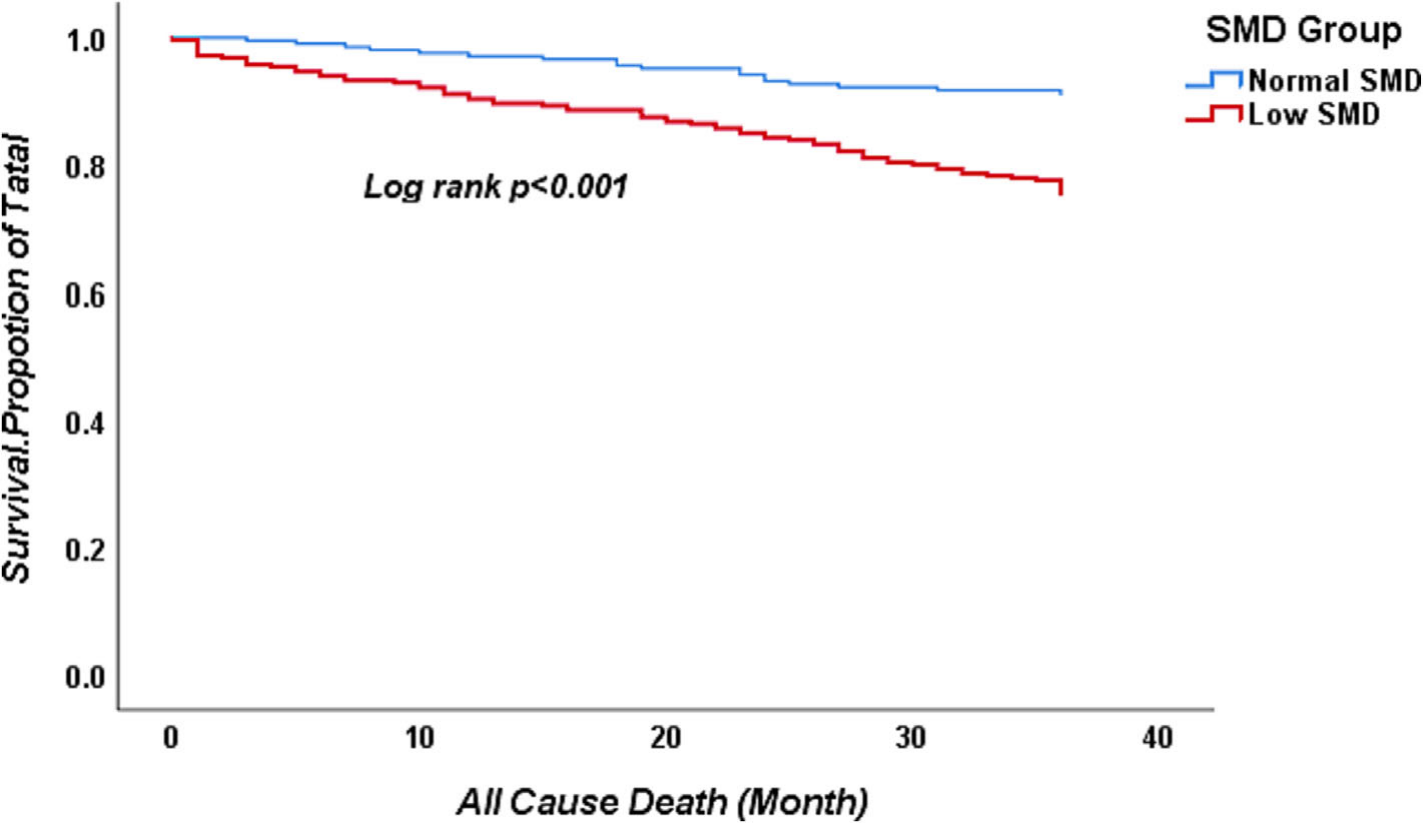
Kaplan–Meier survival curve for overall survival stratified by L1 SMD. Patient survival was compared between two groups that divided by cutoff points. ‘Low SMD (red)’ presents SMD < 39.56 Hounsfield units (HU) for males and <33.06 HU for females. ‘Normal SMD (blue)’ presents SMD ≥ 39.56 HU for males and ≥33.06 HU for females.

**Figure 4:**
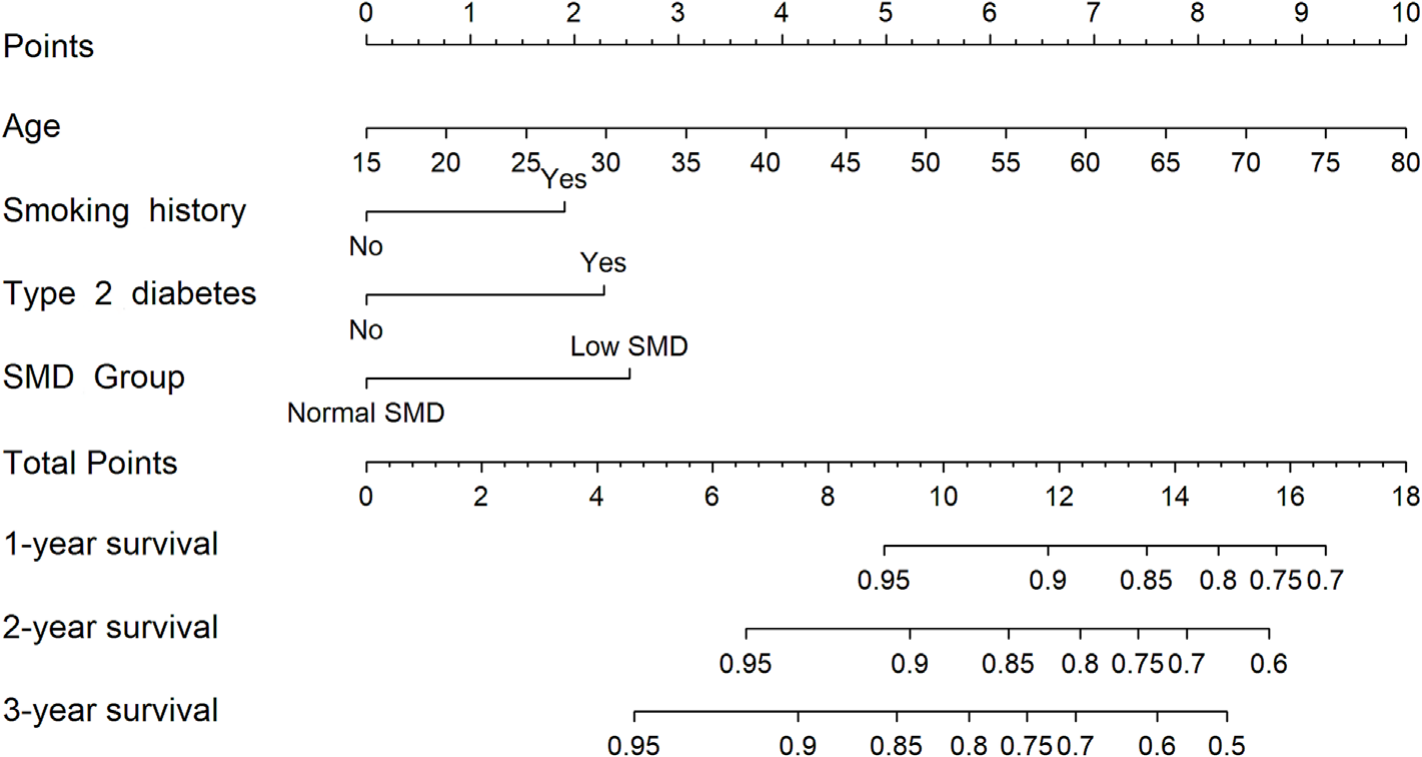
Construction of L1 SMD‐based nomograms in initial‐dialysis patients. ‘Low SMD’ presents SMD < 39.56 Hounsfield units (HU) for males and <33.06 HU for females. ‘Normal SMD’ presents SMD ≥ 39.56 HU for males and ≥33.06 HU for females.

Kaplan–Meier survival analysis for SMD is shown in *Figure* 3. Compared with patients who had normal SMD, patients with low SMD had poor overall survival at each time point (*P* < 0.001). Factors based on multivariate Cox regression analysis with *P* < 0.05 were used to build the nomogram. By adding the score of different risk factors, the total score could be calculated, and then 1‐, 2‐, and 3‐year survival rates were obtained (*Figure* 4).

## Discussion

Sarcopenia is highly prevalent among dialysis patients and causes poor prognoses, such as increased mortality.^10^ However, there is no uniform and accepted diagnostic criterion for sarcopenia.^22^ Our study focused on assessing skeletal muscle mass and quality. To the best of our knowledge, our investigation is the first multicentre retrospective study to apply CT‐assessed L1 to analyse the associations of skeletal muscle mass and quality with mortality in initial‐dialysis patients. In this study, we found high agreement between the L1 and L3 levels of SMI and SMD. We investigated that low L1 SMD, assessed from clinically opportunistic CT images at initial dialysis, may be an objective and reliable risk marker for all‐cause death and cardiac death.

According to previous reports, methods such as dual‐energy X‐ray absorptiometry, bioimpedance analysis, ultrasonic examination, magnetic resonance imaging (MRI) and CT can be chosen to measure skeletal muscle composition.^1^ Compared with CT, ultrasonic examination has some limitations, such as more subjectivity and poor repeatability.^23^ Bioimpedance analysis and dual‐energy X‐ray absorptiometry cannot measure a specific body component directly and can be influenced by fluid overload and residual renal function.^24^ MRI is expensive and has extra contraindications (such as pacemakers).^25^ Additionally, Park et al. found that CT may provide a clearer anatomical boundary of skeletal muscle than MRI, which made it a more reliable skeletal muscle quantification strategy.^26^ Therefore, for dialysis patients, an advantage of our study was that measuring skeletal muscle mass and quality by opportunistic CT can provide a non‐invasive quantification of muscle through high‐quality images, accurate location and precise distinction.^27^

Generally, three kinds of CT scans, namely, chest (including T1 to L1), abdominal (including T10 to L4) and pelvic (including L4 to L5) scans, can be applied for measuring skeletal muscle.^12^ Analysis of a single abdominal CT section at the L3 level has been most frequently performed,^21^ and CT‐assessed L3 SMD has been increasingly recognized as a predictor of mortality in patients with cancer^4^, ^5^, ^6^ and non‐cancer diseases.^7^, ^8^ In the dialysis population, previous studies also mainly used CT images at the L3 level.^16^, ^17^, ^28^ Due to the high morbidity of infection and heart and lung disease in dialysis patients,^29^ chest CT is commonly employed. Especially in the global context of the coronavirus disease 2019 (COVID‐19) pandemic, the frequency of chest CT has increased,^30^ which then expands the clinical application of L1 and does not cause additional expenditure. Considering that images at the L1 level can be more available from opportunistic chest CT than L3 images in dialysis patients, our study compared CT‐assessed SMI and SMD at L1 and L3 and found high agreement between L1 and L3 (*P* < 0.001). The muscle compositions of the L1 and L3 levels are similar (including the psoas major, erector spinae, psoas quadratus, transverse abdomen, internal oblique, external oblique and rectus abdominis), but the fibre type compositions are different, which may explain the differences and similarities between them to some extent.^31^ Similarly, Derstine et al. assessed sarcopenia by T10 to L5 levels in the healthy US population and demonstrated that the L1 level was a good substitute for the L3 level.^12^ In addition, Pickhardt et al. found that the assessment of L1 instead of L3 muscle mass and density was better for predicting death.^13^

Skeletal muscle mass and quality can be assessed by cross‐sectional skeletal muscle area and skeletal muscle density.^11^ Yajima et al. found that CT‐measured lower psoas muscle index and lower psoas muscle density at L3 were independently associated with an increased risk of all‐cause mortality in patients undergoing HD (>6 months).^32^ Recently, some studies found that muscle density instead of muscle mass may represent a more clinically meaningful surrogate of muscle function.^13^, ^33^ Our findings in initial‐dialysis patients were in line with previous studies of cancer patients^4^, ^5^, ^6^ and non‐tumour patients,^34^, ^35^ which supported that low SMD instead of SMI at L1 was associated with a higher risk of mortality. The cardiac mortality rate of dialysis patients (accounting for 52% of all‐cause mortality) was comparable with the cardiac mortality rate (HD: 42.5%, PD: 41.6%) reported by the United States Renal Data System in 2021.^36^ The cardiac mortality rate of the low SMD group (accounting for 58.57% of all‐cause mortality) was higher than that of the normal SMD group. Lee et al. suggested that accumulated fat tissue (indicating lower SMD) was associated with an increased incidence of cardiovascular events.^37^ Kato et al. indicated that carotid artery intima‐media thickness was associated with accumulated abdominal visceral fat, which likely contributed to the increased risk of cardiac death in patients with low SMD.^38^ However, the exact mechanism of low SMD and mortality remains unclear. Low SMD is a manifestation of increased fat infiltration into muscle and is associated with intramuscular lipid deposition.^20^ Some studies have also suggested that low SMD is associated with disease severity, which might be a result of insulin resistance, proinflammatory mediators (such as IL‐6 and TNF‐α), vitamin D deficiency and metabolic acidosis.^39^ Interestingly, we observed the patients in the low SMD group had a relatively high BMI, indicating that patients in this group may suffer from sarcopenic obesity.^40^

The ROC curve proved the significance of the SMD for prognosis, and the nomograms were beneficial for risk stratification. This demands early and effective measures to improve the quality of muscle for long‐term survival. As reported, nutritional therapy (such as protein and vitamin D) combined with exercise may be a valuable approach.^41^

Although our research demonstrated the correlation of L1 and L3 and the association of L1 SMD and mortality in initial‐dialysis patients, there are limitations. First, as a retrospective study, patient information, such as physical activities and dietary patterns, was not available in the EMR. Second, the sample size of patients who had L3 images was small (*n* = 257), and the correlation between L1 SMD and L3 SMD needs further validation in separate cohorts.

## Conclusions

In summary, this study demonstrated high agreements of SMI and SMD between the L1 and L3 levels and revealed that as a skeletal muscle quality indicator measured by opportunistic CT scans, low L1 SMD was associated with a higher risk of all‐cause death and cardiac death in initial‐dialysis patients.

## Conflict of interest

The authors declare that they have no relevant conflicts of interest.

## Funding

This study was supported by grants from the National Natural Science Foundation of China (No. 82230022, 82070735, 82241047 and 82100721), China Postdoctoral Science Foundation (2022M710686), the Foundation of Jiangsu Commission of Health (M2021048), the Project of Taizhou Clinical Medical School of Nanjing Medical University (TZKY20220209), the Young Talent Development Plan of Changzhou Health Commission (2020‐028), the 333 Personnel Training Project of Jiangsu Province (BRA2020156), the Science and Technology Support (Social Development) Project of Bureau of Science and Technology of Changzhou (CE20215024) and the ‘Zhishan Young Scholar’ grants from the Southeast University.

## Supporting information


**Figure S1.** Measurement of the skeletal muscle index and skeletal muscle density.
**Figure S2.** Time‐dependent receiver operating characteristic (ROC) curve of L1 level for dialysis patients based on all‐cause death.
