# Supplementary material for: Computed tomography‐determined skeletal muscle density predicts 3‐year mortality in initial‐dialysis patients in China
Source: J Cachexia Sarcopenia Muscle. 2023 Sep 18;14(6):2569–78. doi: 10.1002/jcsm.13331 (PMC10751407; doi:10.1002/jcsm.13331)
Supplement: Supplementary file 1 — Figure S1. Measurement of the skeletal muscle index and skeletal muscle density. Figure S2. Time‐dependent receiver operating characteristic (ROC) curve of L1 level for dialysis patients based on all‐cause death. [file JCSM-14-2569-s001.docx]

**SUPPLEMENTARY MATERIALS**

**Supplemental Figure 1.** Measurement of the skeletal muscle index and skeletal muscle density

**Supplemental Figure 2.** Time-dependent receiver operating characteristic (ROC) curve of L1 level for dialysis patients based on all-cause death

**
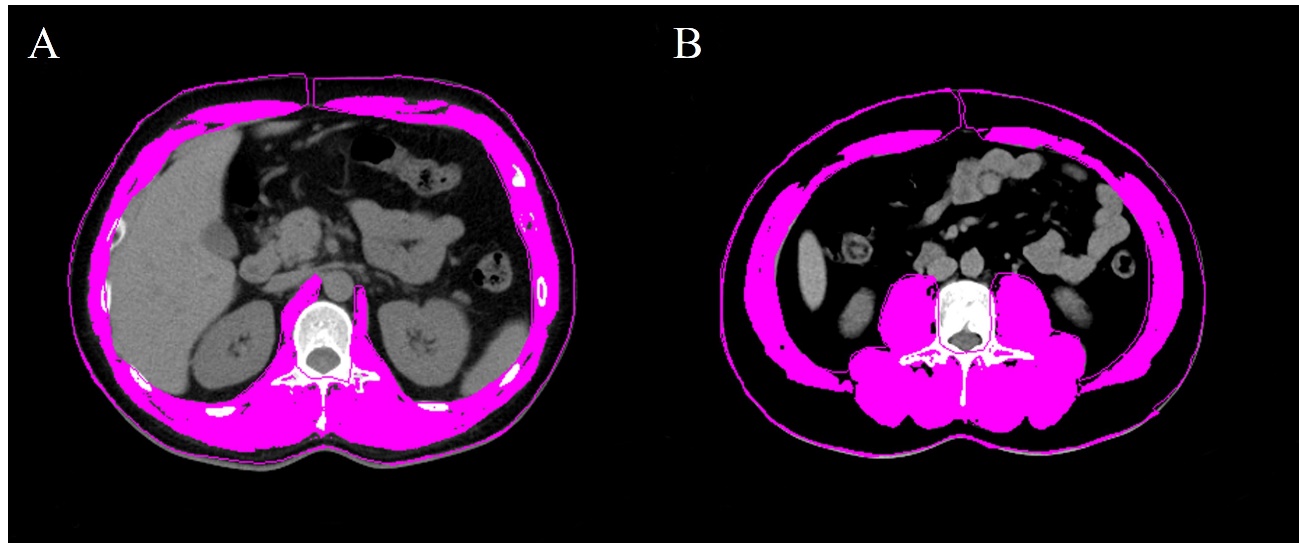
**

**Supplemental Figure 1. Measurement of the skeletal muscle index and skeletal muscle density.** Skeletal muscle areas (purple) were measured using computed tomography images at (A) the first lumbar level, and (B) the third lumbar level.

**
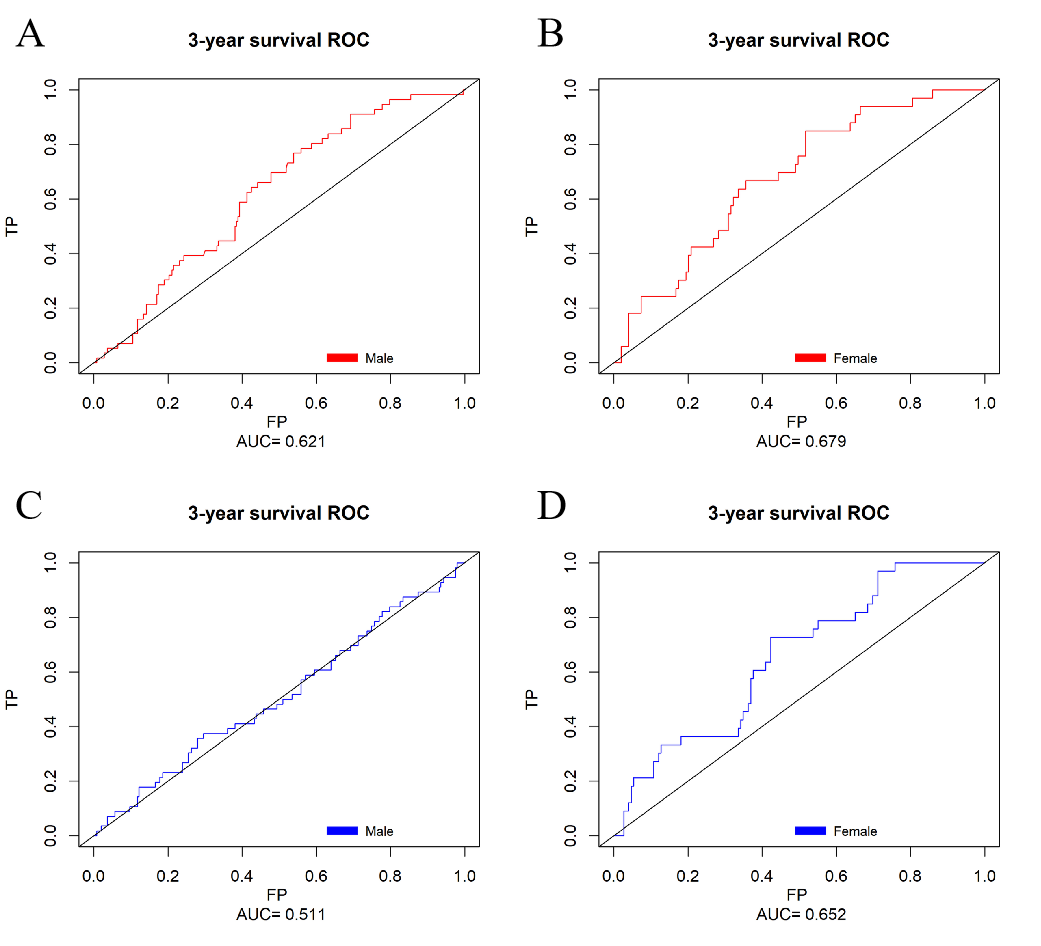
**

**Supplemental Figure 2. Time-dependent receiver operating characteristic (ROC) curve of L1 level for dialysis patients based on all-cause death.** (A). For L1 skeletal muscle density (SMD) of males, the area under the ROC curve was 0.621 and the cutoff value was 39.56 HU (*P* < 0.001), and the sensitivity and specificity were 76.8% and 46.2%, respectively. (B). For L1 SMD of females, the area under the ROC curve was 0.679 and the cutoff value for females was 33.06 HU (*P* < 0.001), and the sensitivity and specificity were 84.8% and 48.3%, respectively. (C). There was no significant association between L1 skeletal muscle index (SMI) of males and all-cause death (*P* > 0.05). (D). For L1 SMI of females, the area under the ROC curve was 0.652 and the cutoff value was 35.25 cm^2^/m^2^ (*P* < 0.001), and the sensitivity and specificity were 72.7% and 57.7%, respectively.
